# Exploiting immune-dependent effects of microtubule-targeting agents to improve efficacy and tolerability of cancer treatment

**DOI:** 10.1038/s41419-020-2567-0

**Published:** 2020-05-12

**Authors:** Angela Flavia Serpico, Roberta Visconti, Domenico Grieco

**Affiliations:** 1 CEINGE Biotecnologie Avanzate, Naples, Italy; 2 DMMBM, University of Naples “Federico II”, Naples, Italy; 3 IEOS, CNR, Naples, Italy; 4 Department of Pharmacy, University of Naples “Federico II”, Naples, Italy

**Keywords:** Medical research, Clinical trial design

## Abstract

Microtubule-targeting agents (MTAs), like taxanes and vinca alkaloids, are tubulin-binding drugs that are very effective in the treatment of various types of cancers. In cell cultures, these drugs appear to affect assembly of the mitotic spindle and to delay progression through mitosis and this correlates with their ability to induce cell death. Their clinical efficacy is, however, limited by resistance and toxicity. For these reasons, other spindle-targeting drugs, affecting proteins such as certain kinesins like Eg5 and CENP-E, or kinases like Plk1, Aurora A and B, have been developed as an alternative to MTAs. However, these attempts have disappointed in the clinic since these drugs show poor anticancer activity and toxicity ahead of positive effects. In addition, whether efficacy of MTAs in cancer treatment is solely due to their ability to delay mitosis progression remains controversial. Here we discuss recent findings indicating that the taxane paclitaxel can promote a proinflammatory response by activation of innate immunity. We further describe how this can help adaptive antitumor immune response and suggest, on this basis and on the recent success of immune checkpoint inhibitors in cancer treatment, that a combination therapy based on low doses of taxanes and immune checkpoint inhibitors may be of high clinical advantage in terms of wide applicability, reduced toxicity, and increased antitumor response.

## Introduction

MTAs have been introduced in cancer therapy since several years and are still among the most widely used antitumor drugs, utilized alone or in combination with other antiblastic drugs, to treat different cancers^1–3^. In addition, MTAs are still an essential resource as second line treatments and for the treatment of tumors that lack known specific molecular targets and cannot benefit from recent advances in targeted therapy. Indeed, the taxane docetaxel has been approved for treatment of castration resistant prostate cancer and of triple-negative breast cancer, alone or in combination with other drugs^4^.

**Fig. 1:**
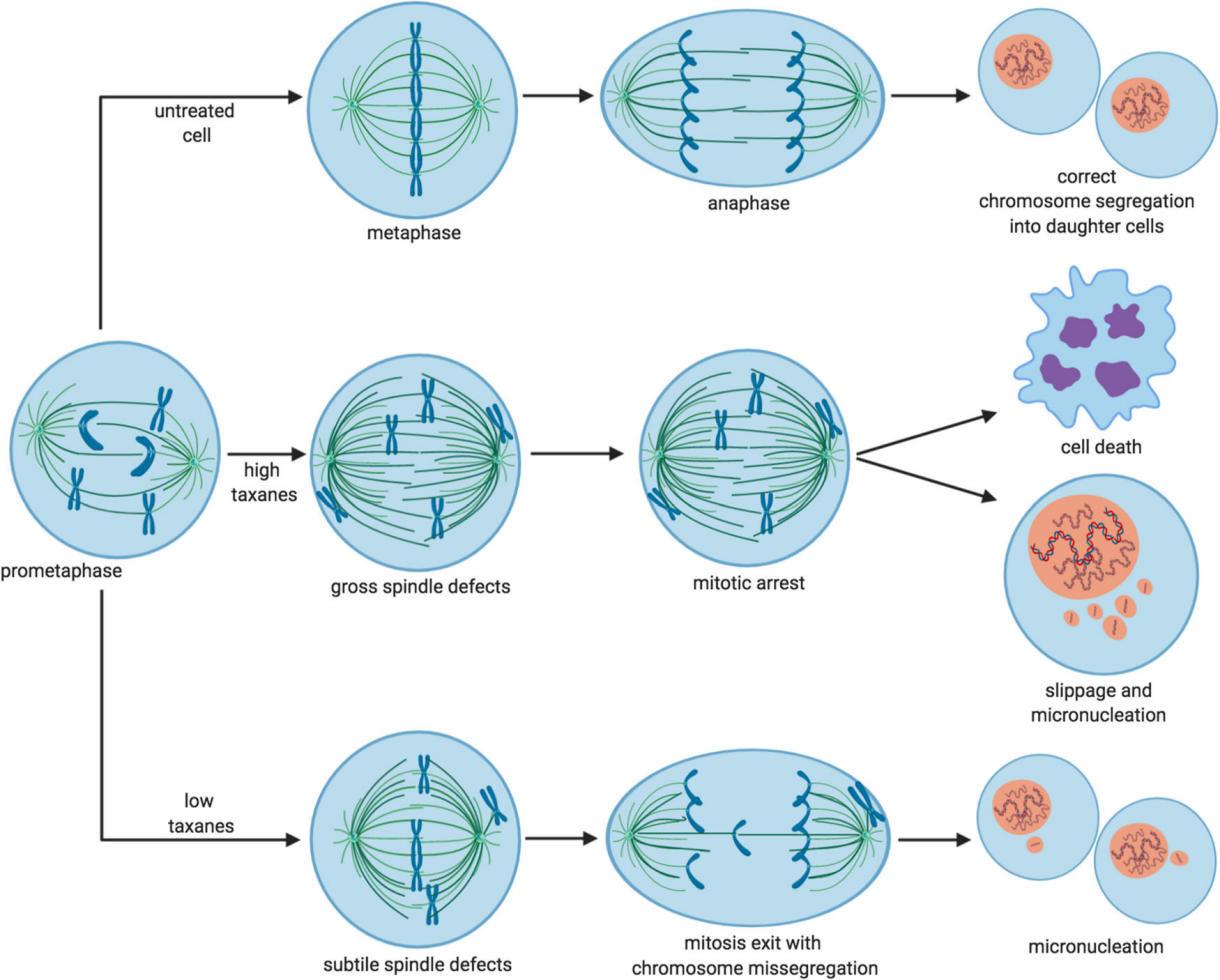
Dose-dependent effects of taxanes on mitosis execution. Top panels; normal chromosome segregation (untreated cells). Middle panels; at high doses (high taxanes), taxanes activate the SAC and delay mitosis exit. This translates in activation of apoptotic pathways that lead to cell death. Nevertheless, cancer cells can escape cell death induced by high taxanes and slip through mitosis without dividing but forming micronuclei (see main text). Lower panels; at low doses (low taxanes), taxanes do not induce significant delay in mitosis exit but rather induce chromosome segregation errors (mitosis exit with chromosome missegregation) and the formation of micronuclei in daughter cells.

MTAs bind *β*-tubulin and severely affect microtubule dynamics through different mechanisms. Taxanes stabilize microtubules while vinca alkaloids hamper microtubule polymerization. Thus, MTAs interfere with many key cellular processes. In interphase, the intracellular transport of proteins, vesicles, and organelles along trucks formed by microtubule fibers are deeply affected by MTAs^5–7^. In mitosis, the microtubular cytoskeleton is profoundly rearranged to form the mitotic spindle, the structure required to segregate replicated chromosomes during cell division, and this is also deeply affected by MTAs^8^. By altering normal mitotic spindle assembly, MTAs activate the spindle assembly checkpoint (SAC), a safeguard mechanism that prevents errors in chromosome segregation and generation of aneuploid cells by delaying mitosis exit when spindle assembly is impaired (Fig. 1; high taxanes)^9,10^. When spindle assembly is incomplete or impaired, SAC effector proteins, like BubR1 and Mad2, bind Cdc20, a coactivator of the ubiquitin ligase Anaphase-Promoting Complex/Cyclosome (APC/C), to form a mitotic checkpoint complex (MCC). MCC inhibits APC/C-dependent degradation and inactivation of the master mitotic kinase cyclin B-dependent kinase (Cdk) 1 and of the anaphase inhibitor securin, arresting cells in mitosis. However, after prolonged SAC-dependent delay in mitosis, cells activate cell-death programs^10–14^. Indeed, in cell cultures, MTA-dependent mitotic perturbance and delayed progression through mitosis correlate with the MTA ability of inducing cell death, thus providing a mechanistic rationale for the therapeutic effects of these drugs. However, as discussed later, mitotic delay may not be the only mechanism by which MTAs kill cancer cells.

## Limitations of MTA-based cancer therapy

In the clinic, most patients immediately respond to treatment with MTAs, with very few cases of naive resistance against these drugs^15^. Unfortunately, patients that initially respond well to MTA therapy may later develop resistance to treatment. The mechanisms of acquired resistance to MTAs are several, spanning from the more general upregulation of the ABC transmembrane efflux transporters to the much more drug specific, “on-target” mutations of microtubule forming or binding proteins. For example, the response to MTAs can be limited by mutations or by altered expression of the microtubule building blocks *α*- and *β*-tubulin^4,15,16^. A more common way of resistance to MTAs, however, is believed to derive from an adaptation mechanism known as “mitotic slippage”^17^. Indeed, even in the presence of therapeutic concentrations of MTAs, cells can override SAC-induced mitotic arrest and slip out of mitosis (Fig. 1; high taxanes). This is mainly due to a progressive loss of Cdk1 activity, during arrest, to a point in which the SAC, that requires Cdk1 activity, cannot be held active anymore in preventing cyclin B degradation and mitosis exit^18,19^. MTA-treated cells can survive if they slip through mitosis before the threshold necessary to induce mitotic delay-dependent cell death has been reached and either die afterwards, or stop proliferating, or give rise to more aggressive clones^8,14^. It can, therefore, be inferred that preventing mitotic slippage for a time sufficient enough to induce mitotic cell-death pathways could avoid this mechanism of resistance. To this end, it has been suggested that inhibiting APC/C^Cdc20^, the ubiquitin ligase responsible for cyclin B and securin degradation and, therefore, crucial for mitosis exit, might help cancer cell killing in aid to MTA treatments^18^. However, the two so far described APC/C^Cdc20^ inhibitors, TAME and Apcin, though promisingly efficient in blocking mitosis exit in experimental settings, are not yet available for clinical use and very recent observations cast doubts on their mechanism of action^20–22^. As an alternative to sustain MTA-induced mitotic arrest, we have proposed to combine MTAs with the Wee1 kinase inhibitor AZD-1775, based on a novel role we unveiled for Wee1 in regulating mitosis exit^23,24^. Of note, we proved that Wee1 genetic depletion substantially delayed mitotic exit and that chemically inhibiting Wee1 with AZD-1775 synergized with MTAs by further prolonging mitosis and increasing cell death in cancer cell lines and primary human lymphoblastic leukemia cells^18,23^.

Besides resistance, the use of MTAs can be substantially limited also by side effects, in some cases so severe to force to dose reduction or discontinuation. Above all, MTAs cause neutropenia and lymphopenia, a consequence of toxicity on cycling hematopoietic precursor cells, and neurotoxicity, likely by disruption of microtubule-mediated axonal transport in neurons^25^. To control this, new MTA formulations have been developed and tested to reduce doses, optimizing delivery, and distribution. The nanoparticle albumin-bound (nab)-paclitaxel is a solvent-free, colloidal suspension of the taxane paclitaxel, and human serum albumin, already approved for cancer therapy^26^. Microspheres and liposomes are currently tested as MTA vehicles^27^.

Looking for an alternative way to delay mitosis exit and promote cancer cell death, many spindle-targeting drugs, not directly targeting tubulin but rather microtubule-associated proteins or kinases required for mitotic spindle assembly, have been developed^28^. Among them, several inhibitors of the kinesin superfamily proteins (KIFs) have been exploited. Kinesins and kinesin-related proteins make up a large superfamily of molecular motors responsible for the major microtubule- and ATP-dependent transport pathways and some of them are particularly relevant for spindle assembly^29,30^. Disappointingly, however, most of mitotic KIF inhibitors tested, although able to perturb mitotic spindle assembly, do not kill cells efficiently^29,30^. A large number of molecules has also been developed to inhibit kinases like Plk1, Aurora A and Aurora B that are required for spindle assembly^31^. Nevertheless, initial clinical trials with most of the Plk and Aurora inhibitors have not confirmed the promising preclinical efficacy and only very few drugs, such as the Aurora A kinase inhibitor alisertib, reached phase III trials for a wide variety of tumors, upon encouraging response rates in phase II trial. However, the positive results have been poorly confirmed in further trials^32,33^.

## Immunotherapies targeting immune checkpoints

Cancers are often infiltrated by a heterogeneous population of tumor-infiltrating immune cells and whether this produces pro- or antitumor effects is still matter of extensive investigation. IFN-*γ*-producing CD4^+^ T helper (Th)1 cells, CD8^+^ T cells, natural killer (NK) cells, type 1 NK T cells, mature dendritic cells (DCs), and M1 macrophages could generate an antitumor response. On the other hand, CD4^+^ Th2 cells, CD4^+^ T regulatory (TReg) cells, type 2 NK T cells, myeloid-derived suppressor cells, immature DCs, and M2 macrophages could suppress antitumor immunity and promote cancer progression^34,35^. T cells are also negatively controlled by immune checkpoint proteins, classes of molecules and signals that restrain T-cell proliferation, survival, and activation^36^. Although cancer cells can express tumor-specific neoantigens, thus being susceptible to be targeted by the immune system, cancer cells often express on their surface immune checkpoint molecules that suppress activation of T cells that could grant tumor immune surveillance. These immune checkpoint molecules, like Programmed cell-Death-1 Ligand (PD-L1) and B7, are normally found on the antigen presenting cell (APC) to avoid auto-immune T-cell activation in the body (T cells are expressing the relative receptors PD-1 and CTLA-4). Based on these observations, immunotherapies targeting these molecules have been developed and are emerging as a major breakthrough in cancer treatment^36,37^.

CTLA-4 is expressed on the cell membrane of T cells and competes with the TCR-costimulatory protein CD28 for binding to the B7 protein of the APC. Upregulation of CTLA-4 expression and increased CTLA-4:B7 binding results in a negative signal, which limits proliferation and survival of the T cells^38^. The exact mechanism by which anti-CTLA-4 antibodies induce an antitumor response is still imprecisely known, although preclinical evidence suggests that CTLA-4 blockade supports the activation and proliferation of a higher number of effector T cells and reduces TReg cell-mediated suppression of effector T-cell response^39,40^. Indeed, after successful clinical trials, the anti-CTLA-4 monoclonal antibody ipilimumab was first approved for the treatment of advanced or unresectable melanoma^41,42^.

PD-1, a cell surface receptor, is expressed on regulatory and cytotoxic activated T cells in peripheral tissues while PD-L1 is mainly expressed on APC. Physiologically, binding of PD-L1 to its receptor results in T-cell inactivation. Thus, PD-1/PD-L1 is an immune checkpoint that guards against autoimmunity, promoting self-tolerance, and is crucial to limit immune responses in case of infections^43,44^. PD-1 is highly expressed on many tumor-infiltrating lymphocytes and cancer cells often overexpress PD-L1, thus escaping immune surveillance^45^. This has provided a strong rationale for the development of drugs targeting the PD-1/PD-L1 checkpoint. Indeed, antibodies blocking the binding of PD-L1 to its receptor, such as nivolumab and pembrolizumab, enhance immunity against a wide variety of cancers^37,46^. FDA rapidly approved these drugs for the treatment of melanoma, urothelial cancer, renal cell carcinoma, non-small-cell lung cancer (NSCLC), Hodgkin lymphoma, and squamous cell carcinoma of head and neck.

Unfortunately, not all cancers respond to Immune Checkpoint Inhibitor (ICI)-based therapy^47^. This appears to correlate very strongly with the relatively poor infiltrate of immune and inflammatory cells of ICI-resistant cancers that are, therefore, called “cold tumors”^48^.

## Combination of immune checkpoints targeting immunotherapies with DNA-damaging treatments

A possible strategy to improve ICI-based therapy in cancer patients is to combine it with radiation or traditional, DNA-damaging, antiblastic therapies. Indeed, by induction of necrosis or immunogenic cell death (ICD), DNA damage may render cold tumors inflamed^49^. It was soon hypothesized that the combination of DNA-damaging radiations or drugs with ICIs could be highly beneficial to cancer patients. Increasing evidence suggests that the antitumor activity of DNA-damaging treatments is mediated not only through cytotoxic effects, but also because they stimulate immune surveillance by affecting both cancer and immune cells^49^.

For example, some DNA-alkylating agents, like cyclophosphamide and carboplatin, or antimetabolites, like pemetrexed, both increase the expression of MHC class I molecules on cancer cells and subvert the immunosuppressive functions of TReg cells^50,51^. The autocrine and paracrine circuits controlling cancer immune surveillance mainly depend on type I IFNs secreted by tumor cells and/or by tumor-infiltrating immune cells^52^. Recently, it has been demonstrated that DNA damaged cancer cells are an important source of type I IFNs. Cells with double-stranded DNA breaks that progress through mitosis accumulate micronuclei^53,54^. Micronuclear DNA is sensed by cGAS, an enzyme that catalyzes the formation of cyclic GMP-AMP (cGAMP) from ATP and GTP^53,54^. cGAMP, upon binding to the adaptor protein Stimulator of Interferon Genes (STING), activates the transcription factor IRF3, leading to the transcription of Type I interferons (IFNs) and, in turn, to an innate immunity response^53–55^. Remarkably, using a well-described B16 syngenic mouse model of melanoma, it has been shown that irradiation of one tumor along with immune checkpoint blockade results in a T-cell-dependent growth delay of a contralateral unirradiated tumor^53^. The irradiated tumor produces cGAS/STING-dependent immunomodulatory signals that result in an efficient immune-mediated regression of the contralateral tumor provided that PD-1/PD-L1- or CTLA-4-mediated signaling are inhibited. The results obtained in the melanoma mouse model, therefore, suggest that the immune checkpoint manipulation could indeed enhance the response to DNA-damaging, radio- and chemotherapies. Indeed, upon successful conclusion of a phase II clinical trial, one such combination treatment (pembrolizumab plus carboplatin/pemetrexed), has been already approved for NSCLC patients^56^. In any case, limitations of combining DNA-damaging radiations or drugs with ICIs appear essentially due to lack of ICI effects because of damage to immune cells by the DNA-damaging agents or, conversely, to adverse, toxic, effects of hyperactivation of inflammatory, and immunological reaction towards normal tissues^57,58^.

## Exploiting immune-dependent effects of MTAs to improve cancer treatment

The fact that most of the spindle-targeting drugs are not so efficacious in cancer treatment has reinforced the idea that MTAs also act independently of their ability to delay mitosis completion^13^. How MTAs might kill cells besides their ability to induce mitotic cell death, is, however, mechanistically unclear and object of great debate^8,13,14,59–61^. Recent observations have indicated that when cells slip through mitosis, after mitotic delay, and adapt to paclitaxel, they form micronuclei that, as in the case of DNA-damaging treatments, activate the cGAS/STING pathway and stimulate a proinflammatory response (Fig. 1, high taxanes)^8,14,62^. Thus, as recently suggested, taxane-based therapy may also work because it elicits the antitumor intervention of the immune system on cells escaping mitotic death^8,63^. Indeed, it has been shown that paclitaxel stimulates breast cancer cells to produce IFN-*β* and taxane-based therapy often induces increased tumor-infiltrating immune cells, despite their suppressive effect on the rapidly cycling bone marrow cells^3,5,64,65^. Thus, taxane-based therapies could benefit by the combination with ICIs. Several clinical trials have also been designed now to explore the effect of a combinatorial therapy with taxanes and ICIs. The majority of these clinical trials are still ongoing and their preliminary but very promising results are still to be definitively proven. It is a fact, however, that upon successful completion of two such trials, pembrolizumab and atezolizumab, another anti-PD-L1 monoclonal antibody, have been approved in combination with paclitaxel or its albumin-stabilized nanoparticle formulation nab-paclitaxel for the first-line treatment of metastatic squamous NSCLC^66,67^. Moreover, atezolizumab in combination with the sole nab-paclitaxel has also been approved for the treatment of women with unresectable triple-negative breast cancer^68^. The efficacy of the combination of taxanes and ICIs in cancer therapy may be explained by a simple additive effect of the two classes of drugs. However, as already discussed, the complex and not yet completely investigated immunomodulatory activity of MTAs on tumor-infiltrating immune cells might at least in part explain the success of the MTAs and ICIs combination^63^.

## Combination of ICIs with low doses of MTAs

**Fig. 2:**
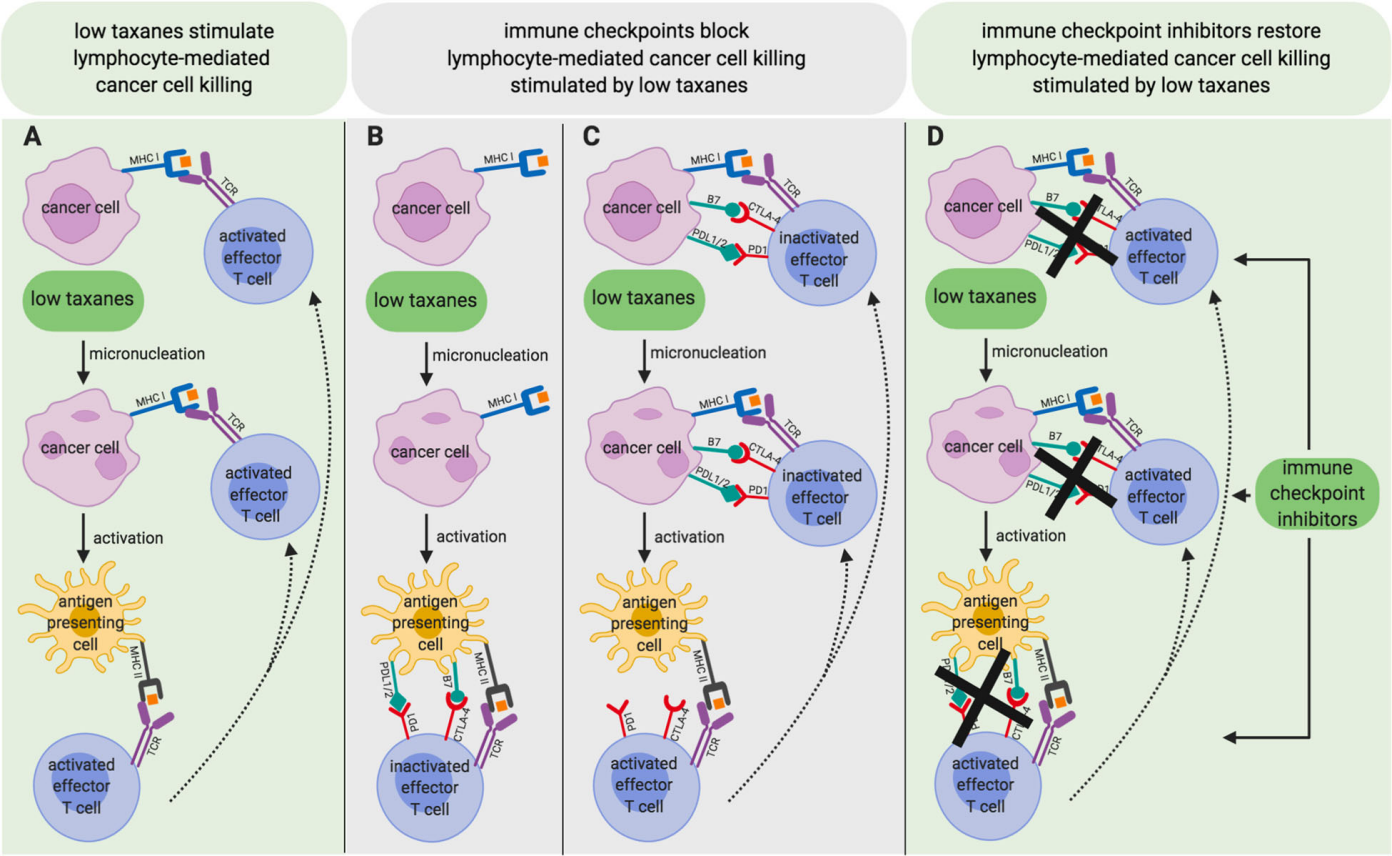
Low taxane-induced micronucleation stimulates innate immunity response and may promote lymphocyte-mediated cancer cell killing when combined with ICI treatment. Cancer cells may express tumor-specific neoantigens and when treated with low doses of taxanes may induce micronucleation-dependent activation of antigen presenting cell (APC). **a** Micronucleation-dependent activation of APC may stimulate adaptive immunity to promote effector T lymphocyte-mediated cancer cell killing. **b** Micronucleation-dependent activation of APC may stimulate adaptive immunity but keep effector T lymphocytes under check by upregulating immune checkpoint molecules (immune checkpoint ligands and cognate receptors are indicated in green and red, respectively). **c** Cancer cells themselves may upregulate immune checkpoint molecules and keep effector T lymphocytes under check. **d** The combination of low doses of taxanes with ICIs unleashes potent effector T-cell-mediated cancer cell killing.

Death after prolonged mitosis or following slippage is certainly a way MTAs kill cancer cells. However, in the case of paclitaxel, recent correlations between clinical therapeutic success for breast cancer patients and the type of mitotic aberrations induced by this drug in their breast cancer cells have indicated that the therapeutic benefit correlates with alterations in chromosome segregation rather than with prolongation of the duration of mitosis^69^. Indeed, while at relatively high doses paclitaxel induces mitotic delay, at much lower concentrations it does not significantly delay mitosis duration but perturbs its normal execution inducing a significant degree of chromosome missegregation and formation of micronuclei in daughter cells (Fig. 1; low taxanes)^70^. When single or small groups of chromosomes do not segregate with the mass of other chromosomes, they become wrapped up in nuclear membranes and remain separate from the primary nucleus^8^. Micronuclei formed upon chromosome segregation errors bear extensive membrane defects because ‘non-core’ nuclear envelope proteins, including nuclear pore complexes, do not assembly properly on lagging chromosomes^71^. Thus, micronuclei spontaneously and frequently lose nuclear envelope integrity, generating further DNA damage^72^. This micronuclear DNA can activate the cGAS-STING pathway stimulating macrophages and innate immunity and, as discussed earlier, innate immunity may help adaptive immunity and favor antitumor immune surveillance (Fig. 2a)^8^.

These observations suggest not only that a major reason for the therapeutic success of taxanes, and perhaps of other classes of MTAs, relies on their ability to promote antitumor immune surveillance but also that low doses of the drugs may be sufficient to achieve this goal. By induction of micronucleation and cGAS/STING-signaling, low doses of taxanes might be sufficient to activate innate immunity and inflammation, giving less negative side effects than standard therapeutic regimens as neutropenia and lymphopenia that may oppose to antitumor immune surveillance^25^. Thus, low doses of taxanes would be sufficient to enhance recruitment of immune cells and render “hot” otherwise “cold” tumors now readily attackable by the immune system (Fig. 2a). However, immune checkpoint may oppose to antitumor immune surveillance stimulated by taxanes (Fig. 2b, c). Based on these considerations, we would like to propose the use of low doses of taxanes in combination with ICIs as a strategy of wide applicability, high tolerability, and efficacy in cancer treatment (Fig. 2d).
